# Malaria Infection Has Spatial, Temporal, and Spatiotemporal Heterogeneity in Unstable Malaria Transmission Areas in Northwest Ethiopia

**DOI:** 10.1371/journal.pone.0079966

**Published:** 2013-11-06

**Authors:** Kassahun Alemu, Alemayehu Worku, Yemane Berhane

**Affiliations:** 1 Department of Environmental and Occupational Health and Safety, Institute of Public Health, College of Medicine and Health Sciences, University of Gondar, Gondar, Ethiopia; 2 Department of Epidemiology and Biostatistics, School of Public Health, College of Health Sciences, Addis Ababa University, Addis Ababa, Ethiopia; 3 Addis Continental Institute of Public Health, Addis Ababa, Ethiopia; National Institute of Medical Research, United Kingdom

## Abstract

**Background:**

Malaria elimination requires successful nationwide control efforts. Detecting the spatiotemporal distribution and mapping high-risk areas are useful to effectively target pockets of malaria endemic regions for interventions.

**Objective:**

The aim of the study was to identify patterns of malaria distribution by space and time in unstable malaria transmission areas in northwest Ethiopia.

**Methods:**

Data were retrieved from the monthly reports stored in the district malaria offices for the period between 2003 and 2012. Eighteen districts in the highland and fringe malaria areas were included and geo-coded for the purpose of this study. The spatial data were created in ArcGIS10 for each district. The Poisson model was used by applying Kulldorff methods using the SaTScan™ software to analyze the purely temporal, spatial and space-time clusters of malaria at a district levels.

**Results:**

The study revealed that malaria case distribution has spatial, temporal, and spatiotemporal heterogeneity in unstable transmission areas. Most likely spatial malaria clusters were detected at Dera, Fogera, Farta, Libokemkem and Misrak Este districts (LLR =197764.1, p<0.001). Significant spatiotemporal malaria clusters were detected at Dera, Fogera, Farta, Libokemkem and Misrak Este districts (LLR=197764.1, p<0.001) between 2003/1/1 and 2012/12/31. A temporal scan statistics identified two high risk periods from 2009/1/1 to 2010/12/31 (LLR=72490.5, p<0.001) and from 2003/1/1 to 2005/12/31 (LLR=26988.7, p<0.001).

**Conclusion:**

In unstable malaria transmission areas, detecting and considering the spatiotemporal heterogeneity would be useful to strengthen malaria control efforts and ultimately achieve elimination.

##  Introduction

Malaria is one of the top priority communicable diseases targeted for elimination by the World Health Organization. It affects a large segment of the population in the malaria vulnerable regions. Children and pregnant women are severely and disproportionately affected by malaria in high malaria burden countries[1]. It remains a major public health problem in Ethiopia where two-thirds of its population lives in malaria transmission areas[2-4]. The transmission shows significant variations in time and space[5,6]. 

Effective and efficient malaria interventions require a good understanding of the epidemiology [7], and transmission dynamics in time and space. Targeting heterogeneity at all levels of transmission intensity could improve malaria intervention strategies and control measures [8-13]. In recent years, technological and scientific advances have created the possibility for doing such elaborate analysis to identify geo-spatial clustering. Thus, efforts must be intensified to use the available data for targeting interventions based on local transmission trends.

In Ethiopia, few spatiotemporal studies of malaria have been reported in recent years [14-16]. Thus, the heterogeneity of malaria transmission is not yet fully explored and the approaches used for detecting the prevailing heterogeneity are different from the approaches used in this study. This study was used scan statistics to study malaria transmission heterogeneity for a variety of reasons. Firstly, the spatial scan statistics method could identify malaria clusters and demonstrate malaria risk heterogeneity at the local level[9]. Secondly, it has enough power to reject the null hypotheses of homogeneous relative risk[17]. Thirdly, it enables the research to provide a description of spatiotemporal heterogeneity, clarify the epidemiology of malaria, prioritize resource allocation, and investigate malaria heterogeneity at fine geographical scale[13]. The aim of this study was thus to detect purely spatial, temporal, and space-time malaria clusters at a district levels in northwest Ethiopia.

## Methods

### Study area

**Figure 1:**
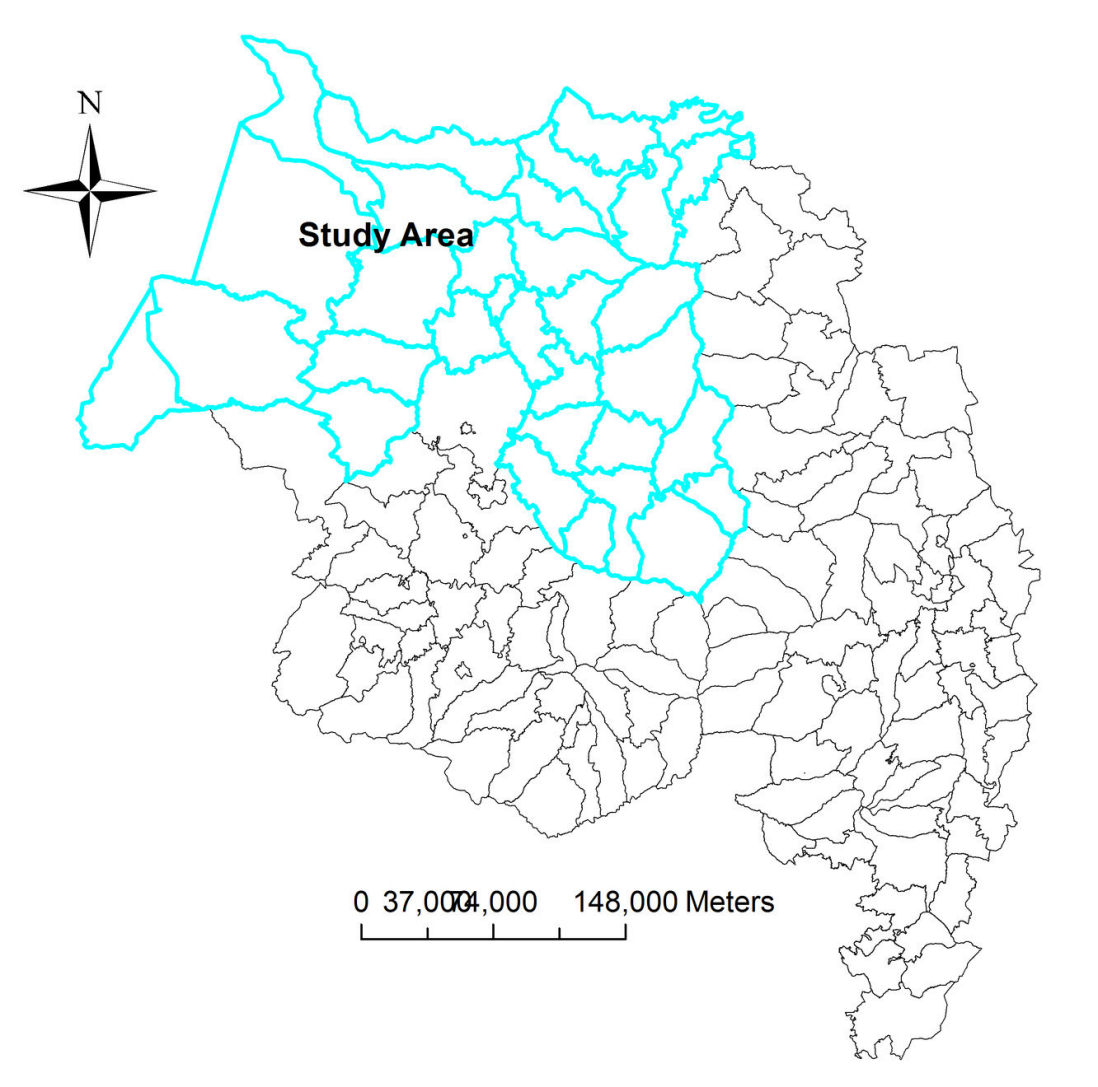
Location of the study area, Amhara national regional state, northwest Ethiopia.

The study was conducted in North and South Gondar administrative zones, in northwest Ethiopia (Figure 1). About 5.6 million people are estimated to live in the area, according to the Central Statistical Agency of Ethiopia (CSA)[18]. The study districts experience a bimodal rainfall pattern; the main rainy season is from June to September followed by a short spring between February and May. Recorded temperatures in the study area showed an average minimum temperature of 12.12°c, an average maximum temperature of 25.4°c, an extremely average minimum temperature of 9.3°c, and an extremely average maximum temperature of 28.3°c. 

Of the 30 districts in the study area, twenty-five are in the highland and fringe regions. In these districts, malaria transmission is unstable, seasonal, and characterized by frequent epidemics. The peak times of malaria transmission occur between September and December (i.e. following the main rainy season from June to August) and from April to June [2,4,19]. Unstable malaria transmission is defined as irregular transmission in highland and fringe areas with substantial yearly and seasonal fluctuations. These areas are prone to malaria epidemic; immunity is generally low and all age groups of the population are at risk of the disease[20]. Thus, districts in the highland and fringe were included in the study.

### Data

Data on malaria were obtained retrospectively from monthly reports to the district health offices between early 2003 and late 2012. The data were reported from health facilities to the district health offices in monthly surveillance forms. The malaria datasets were aggregated at a district levels and comprised information on malaria cases, type of parasites (p.falciparum, p.vivax and mixed infections), and time of illness (month and year).

The spatial coordinates (the latitudes and longitudes) for each district were obtained from the CSA. The spatial data were created in ArcGIS10[21]for each district. An estimated midyear population of each district was extracted from the CSA and combined with the census tract polygon shaped file. The population data were used to calculate annual malaria incidence and used as known underlying population at risk to fit Poisson model[18]. 

### Analysis

The monthly and annual cumulative malaria incidences of each district were calculated and plotted to check the annual fluctuations of malaria transmission between early 2003 and late 2012. The number of malaria cases to population at risk was used to calculate the monthly and annual cumulative malaria incidences during the specified period. 

The auto regressive integrated moving average (ARIMA) model was used to evaluate the seasonal and annual patterns of malaria transmission in the study districts. The seasonal decomposition procedure was performed for a trend analysis to remove a periodic component from a time series and produce a series that was more suitable for trend analysis. An examination of the autocorrelations and partial autocorrelations of the time series were used to determine the underlying periodicity. A multiplicative model was used for the seasonally adjusted series which were multiplied to yield the original series. In effect, the estimated trends showed seasonal components that were proportional to the overall level of the series. 

#### Poisson Model

The discrete Poisson model was used as the number of cases in each location was Poisson distributed and the nature of the data were count [22]. Patients with malaria were taken as cases, and the population was the combined number of person-years lived used to fit the Poisson model. Then, the Poisson data were analyzed with the purely temporal, spatial, and space-time scan statistics.

#### Cluster analysis

The scan statistics developed by kulldorff and SaTScan™ software version 9.1[23] were used to identify the presence of the purely spatial, temporal, and space-time malaria clusters. The scan statistics did scanning gradually across time and/or space to identify the number of observed and expected observations inside the window at each location. The scanning window was an interval (in time), a circle (in space) or a cylinder with a circular base (in space-time) to which window sizes were determined, and the window with the maximum likelihood was the most likely cluster, and a p-value was assigned to this cluster. 

The spatial scan statistics used a circular window variable radius that moved across the map. The window was in turn centered on each of the several possible grid points positioned throughout the study districts. For each grid point, the radius of the window differed continuously in size from zero to specified maximum value. Thus, the circular window was flexible both in location and size. Every circle was a likely candidate cluster. 

The space-time scan statistics were defined by a cylindrical window with a circular geographic base and with height corresponding to time. The base was defined exactly as for the purely spatial scan statistics, whereas the height reflected the time of potential clusters. The cylindrical window was then moved in space and time so that for each potential geographical location and size it also visited each possible time period. In effect, an infinite number of overlaid cylinders of different shapes and sizes were found, together covering the whole study districts, where every cylinder reflected a possible cluster. 

The temporal scan statistics used a window that moved in one dimension, time, defined in the same way as the height of the cylinder used by the space-time scan statistic. This means that it was flexible in both the start and end date. The maximum temporal length was specified on the temporal window tab. 

For each location and size of the scanning window, the alternative hypothesis was that there was an elevated risk within the window as compared to the outside. The likelihood function was maximized over all window locations and sizes, and the one with the maximum likelihood comprised the most likely cluster. This was the cluster that was least likely to have occurred by chance. The likelihood ratio for this window comprised the maximum likelihood ratio test statistic. The p-value was obtained through the Monte Carlo hypothesis testing[24], by comparing the rank of the maximum likelihood from the real datasets with the maximum likelihoods from the random datasets. The number of replications was limited to 999[25]. It was always clear whether to keep or reject the null hypothesis for typical cut-off values at 5% level of significance. 

The scan was used to scan for areas with high rates (clusters). For purely spatial and space-time analyses, secondary clusters were identified in the datasets in addition to the most likely cluster, and were ordered them according to their likelihood ratio test statistic. The inferences of secondary clusters were adjusted for more likely clusters in the data using the iterative manner[24]. In the first iteration, only the most likely cluster was reported. That cluster was then removed from the datasets. In a second iteration, a completely new analysis was conducted using the remaining data. This procedure was then repeated until there were no more clusters with a p-value less than 0.05. The maximum cluster size was set to 50% of the population at risk. For purely temporal analyses, only the most likely cluster was reported.

### Ethical Clearance

The protocol was approved by the Institutional Review Board (IRB) of the University of Gondar. The IRB waived that the research could be done based on record review without contacting patients. Support letters were obtained from local health offices for retrieving retrospective malaria data from records. All the information was kept confidential and no individual identifiers were collected.

## Result

### Distribution and Trends of Malaria Infections

Eleven of the eighteen districts from the unstable highland malaria transmission areas included in this study were from the North Gondar administrative zone while the remaining seven were from South Gondar. About 2.7 million malaria cases were reported from 2003 to 2012. *Plasmodium falciparum* (67.53%) was the dominant species in the area followed by *plasmodium vivax* (25.64%) and mixed infections (6.83%). All districts reported malaria cases during the study period. The highest (64.7%) of the malaria cases were adults and infants accounted for only 3.79% of the total cases. 

**Figure 2:**
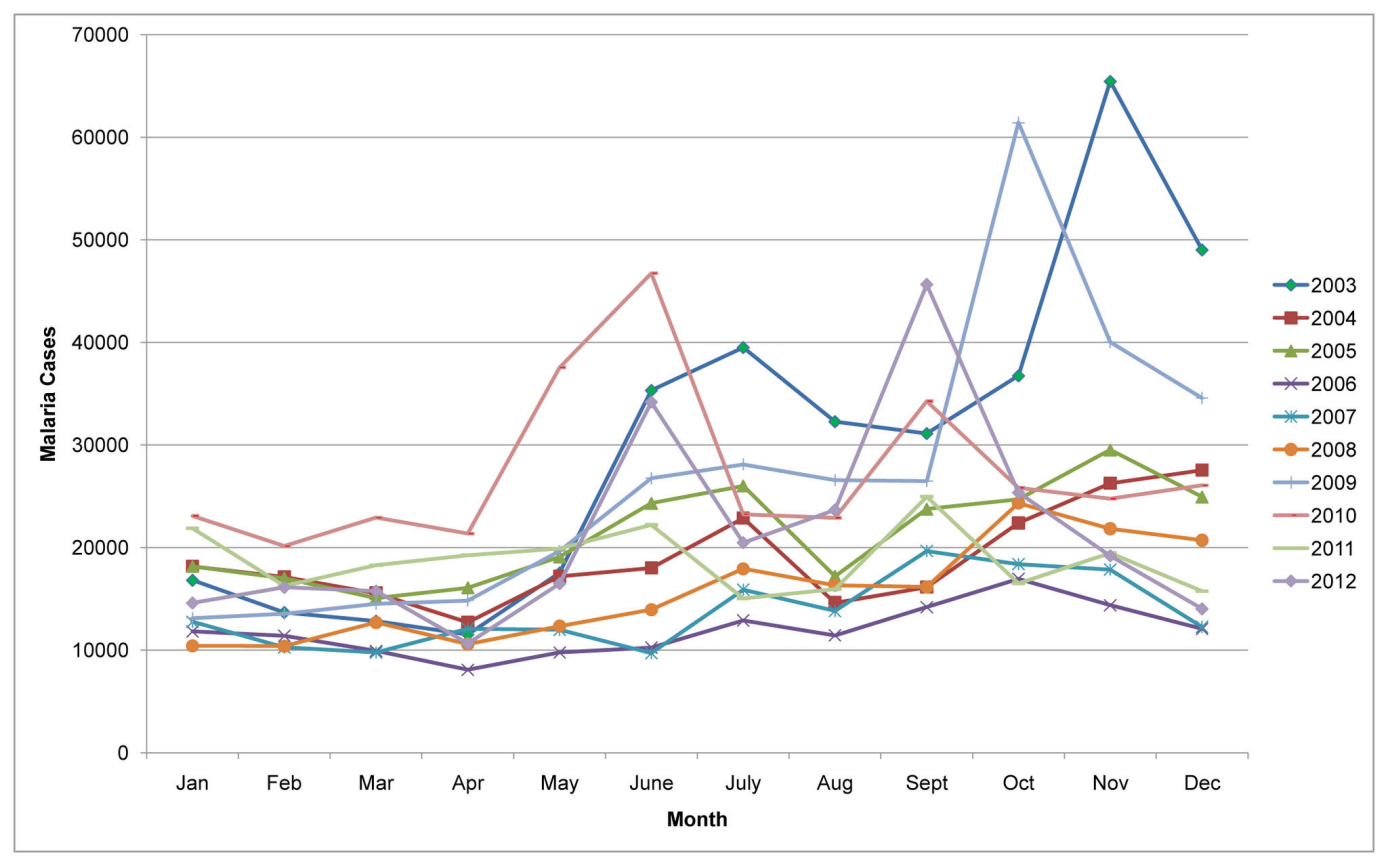
Monthly and seasonal variation of malaria transmission in northwest Ethiopia, between 2003 and 2012. The peak malaria transmission period occurred between September and December, during which nearly 40.6% of the annual malaria cases were reported.

Malaria cases were reported in every month of the year throughout the study period. A seasonal variation of malaria transmission was observed. The main transmission began in mid August and peaked in September and October, declining at the end of November. The second peak of malaria transmission occurred between April and June (Figure 2). An elevated proportion of annual malaria cases (40.8%) were reported between September and December.

**Figure 3:**
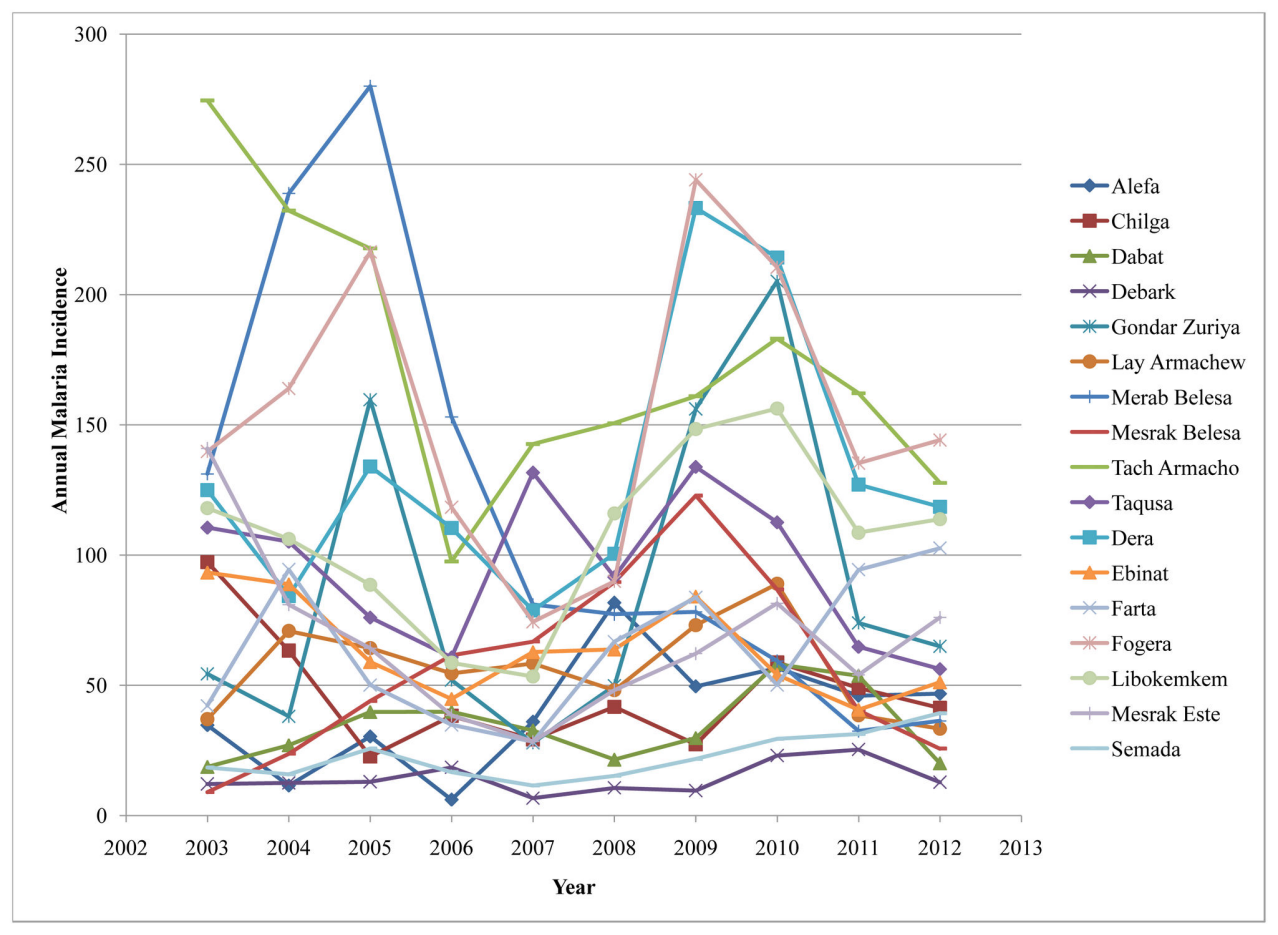
Annual malaria cumulative incidence at the district level in northwest Ethiopia between 2003 and 2012. The highest malaria incidence (244 per 1000 population at risk) occurred in Fogera district in 2009 while the lowest malaria incidence (6.1 per 1000 population at risk) occurred in Alefa district in 2006. Fluctuating temporal trends of annual malaria incidence were observed.

The overall average cumulative annual malaria incidence during the study period was 97 per 1000 population at risk. The highest incidences of malaria cases (244 per 1000 population at risk) occurred in Fogera district in 2009 while the lowest incidence of malaria cases (6.1 per 1000 population at risk) occurred in Alefa district in 2006. Though malaria distribution varied across the districts, most of them had 50 and more malaria cases per 1000 population at risk. During the study period, three malaria epidemic years were observed in 2003, 2009, and 2010 (Figure 3).

**Figure 4:**
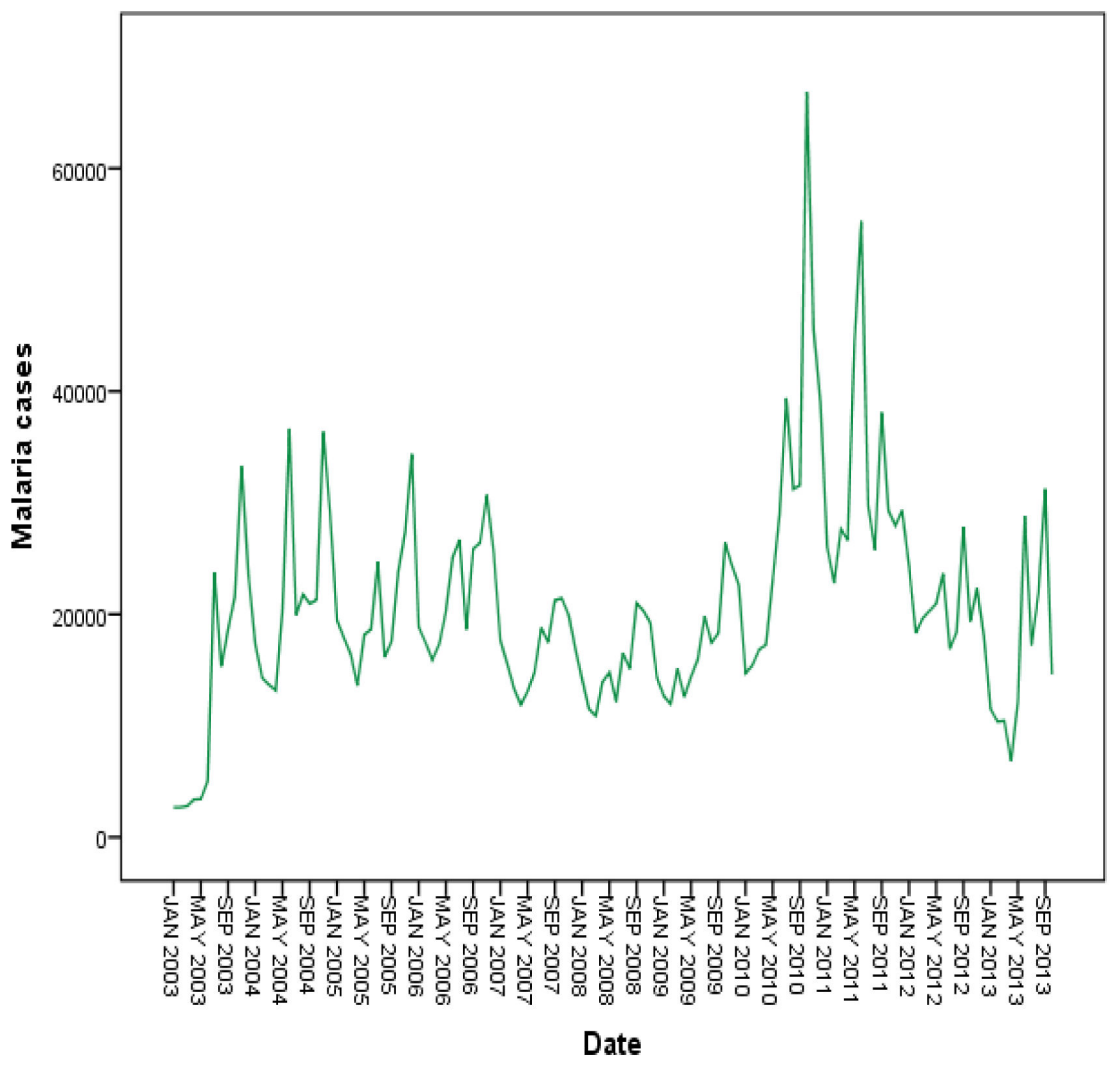
Annually and monthly patterns of malaria transmission between 2003 and 2012 in northwest Ethiopia.

The seasonally adjusted series show that increasing malaria cases occurred between the years 2003 and 2006. A marked decrease in malaria cases were observed in 2007 and 2008. A significant increase was observed over again between 2009 and 2011 and slightly decreased in 2012 (Figure 4). 

### Distribution of High Rate Malaria Spatial Clusters

**Figure 5:**
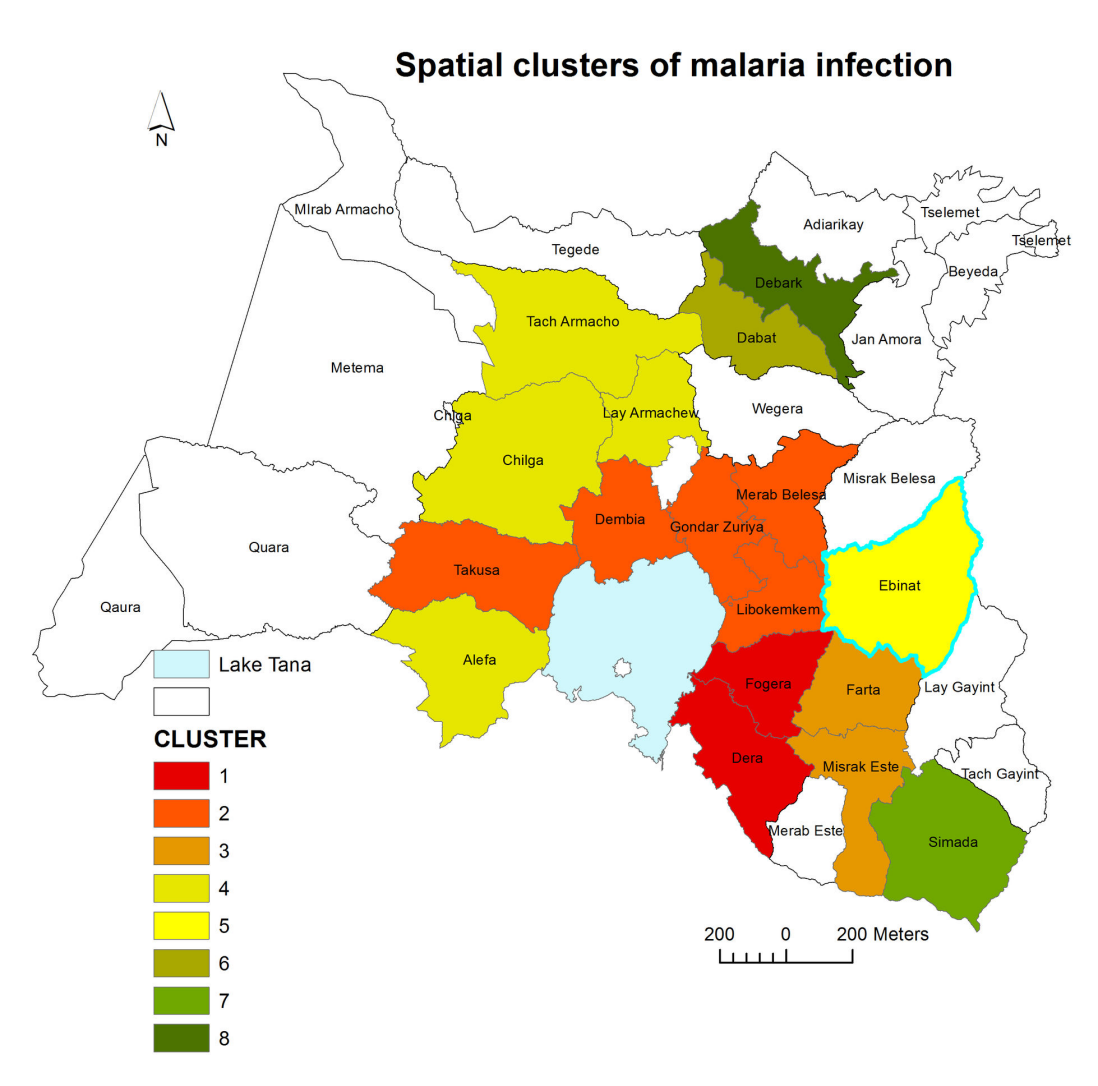
Spatial distribution of significant high rates malaria clusters at a district levels in northwest Ethiopia between 2003 and 2012. Color identification of the clusters was ordered based on the value of likelihood ratio test statistic.

In the study districts, malaria was not distributed randomly. Seven high rate spatial clusters were detected throughout the study period. Dera, Fogera, Farta, Libokemkem and Misrak Este districts (located in South Gondar administrative zone) were the most likely clusters (LLR = 197764.1, p<0.001). Secondary clusters were detected in Dembia, Merab Belesa, and Takusa districts (LLR=167014.6, p<0.001) (Table 1 and Figure 5)

**Table 1:** Significant high rates of spatial clusters of malaria in northwest Ethiopia between 2003 and 2012.

**Cluster**	**District**	**Coordinate/Radius**	**Observed Cases**	**Expected Cases**	**RR**	**LLR**	**P value**
1	Dera	11.6998 N, 37.6248E /49.0km	1227670	772393.8	2.36	197764.1	0.001
1	Fogera	11.9222N,37.6958E/49.0km	1227670	772393.8	2.36	197764.1	0.001
1	Farta	11.6998 N, 37.6249 E/49.0km	1227670	772393.8	2.36	197764.1	0.001
1	Misrak Este	11.6076N,38.0542E/49.0km	1227670	772393.8	2.36	197764.1	0.001
1	Libokemekem	12.1165N,37.7731E/49.0km	1227670	772393.8	2.36	197764.1	0.001
2	Dembia	12.2430N, 37.2921E /26.3km	443523	192612.8	3.47	167014.6	0.001
2	Merab Belesa	12.2800 N,37.4900 E/26.3km	443523	192612.8	3.47	167014.6	0.001
2	Takusa	12.1931N,37.0554E/26.3km	443523	192612.8	3.47	167014.6	0.001
3	Tach Armacho	13.2238N, 37.1488E /60.4km	232371	66184.1	5.74	163154.1	0.001
3	Lay Armacho	12.4459N,37.4157E/80.5km	232371	66184.1	5.74	163154.1	0.001
4	Misrak Belesa	12.4055N, 38.0881E /0km	55148	15934.0	4.12	32563.7	0.001
5	Dabat	13.2238 N, 37.1488E /76.7km	97618	51960.3	2.67	23417.9	0.001
5	Chilga	12.5396N, 37.0587E /76.7km	97618	51960.3	2.67	23417.9	0.001
6	Alefa	11.9286 N, 36.8737E /0 km	43871	21060.8	2.81	12635.5	0.001
7	Semada	11.4092N, 38.2348E /0 km	42466	17148.8	5.24	21497.6	0.001
8	Debark	13.1421N, 37.8979E /0 km	22713	5535.7	3.0	32064.3	0.001

RR=Relative risk; LLR=Log likelihood ratio

### Distribution of High Rate Spatiotemporal Malaria Clusters

 In the study area, significantly high rates of spatiotemporal malaria clusters were identified. Dera, Fogera, Farta, Libokemkem and Misrak Este districts were the most likely spatiotemporal clusters (LLR=197764.1, p<0.001) from 2003/1/1 to 2012/12/31. Secondary clusters were identified in all districts (LLR=72490.5, p<0.001) from 2009/1/1 to 2010/12/31 (Table 2).

**Table 2:** Significant high rates of spatiotemporal clusters of malaria in northwest Ethiopia between 2003 and 2012.

**Cluster**	**District**	**Coordinates /Radius**	**Time Frame**	**Observed Cases**	**Expected Cases**	**RR**	**LLR**	**P value**
1	Dera	11.6998N,37.6248E/49.0km	2003/1/1 to 2012/12/31	1227670	772393.8	2.36	197764.1	0.001
1	Fogera	11.9222N,37.6958E/49.0km	2003/1/1 to 2012/12/31	1227670	772393.8	2.36	197764.1	0.001
1	Farta	11.6998N,37.6249E/49.0km	2003/1/1 to 2012/12/31	1227670	772393.8	2.36	197764.1	0.001
1	Misrak Este	11.6076N,38.0542E/49.0km	2003/1/1 to 2012/12/31	1227670	772393.8	2.36	197764.1	0.001
1	Libokemkem	11.6076N,38.0542E/49.0km	2003/1/1 to 2012/12/31	1227670	772393.8	2.36	197764.1	0.001
2	All	All	2009/1/1 to 2010/12/31	648645	414844.9	1.80	72490.5	0.001
3	Takusa	12.1931N,37.0553E/26.3km	2009/1/1 to 2010/12/31	125778	39776.0	3.30	60564.2	0.001
3	Dembia	12.2430N,37.2922E/26.3km	2009/1/1 to 2010/12/31	125778	39776.0	3.30	60564.2	0.001
4	Tach Armacho	13.2238N, 37.1488E/0km	2003/1/1 to 2012/12/31	145895	53378.5	2.86	56234.4	0.001

RR=Relative risk; LLR=Log likelihood ratio

### Distribution of Temporal Malaria Clusters

In the districts, significantly high rates of purely temporal malaria clusters were observed. Two epidemic years were observed throughout the study period. These anomalies were observed in all districts from 2009/1/1 to 2010/12/31 (LLR=72490.5, p<0.001). The secondary temporal clusters (epidemic years) were identified in all districts from 2003/1/1 to 2005/12/31 (LLR=26988.7, p<0.001) (Table 3). 

**Table 3:** Significant high rates of temporal clusters of malaria in northwest Ethiopia between 2003 and 2012.

**Cluster**	**Districts**	**Time Frame**	**Observed Cases**	**Expected Cases**	**RR**	**LLR**	**P value**
1	All	2009/1/1 to 2010/12/31	648645	414844.9	1.80	72490.5	0.001
2	All	2003/1/1 to 2005/12/31	730456	589405.7	1.46	26988.7	0.001
3	All	2007/1/1 to 2011/12/31	576471	456168.6	1.99	39838.1	0.001

RR=Relative risk; LLR=Log likelihood ratio

## Discussion

The findings of this study show that malaria transmission remained high with occasional large epidemics in space and time in northwest Ethiopia between 2003/1/1 and 2012/12/31. 

The result shows that annual malaria incidences were high in most districts. These areas are under the category of high transmission of malaria. Though rigorous interventions have been carried out by the government and malaria prevention and control partners [3,26], malaria remains a major public health problem in the study districts. 

The spatial cluster analysis indentified high risk districts, which showed the spatial distribution of malaria within unstable highland and fringe areas. The spatial distribution was closely related to the geography of the districts. Most of the high risk districts, like Fogera, Dera, Dembia, Takusa, Alefa, Gondar Zuriya and Libokemkem border with Lake Tana. The peripheries of Lake Tana may contribute to the breeding of the vectors. Thus, this study identified what geographic areas were at the highest risk of malaria, and the clusters identified might have been the areas where malaria prevention and control interventions should be given priority [12]. 

The spatiotemporal cluster analysis identified a high variability of malaria risk over space and time. The most likely spatiotemporal clusters were found at Dera, Fogera, Farta, Libokemkem and Misrak Este districts between 2003/1/1 and 2012/12/31where spatial clusters were identified. This may be due to the fact that the malaria intervention measures might have not been taken appropriately, or the interventions might have not been utilized correctly. This cluster analysis also shows that malaria transmission had significantly gone down from 2006/1/1 to 2008/12/31, after the commencement of the malaria interventions in 2004[26]. However, malaria transmission has been increasing excessively in both space and time in all study districts since 2009/1/1.

The purely temporal cluster analysis detected two high risk periods (epidemics) from 2003/1/1 to 2005/12/31, and 2009/1/1 to 2010/12/31. This shows annual pattern and seasonal variation that exhibits a series of high intensity years with some low levels in between. This implies that seasonal and unstable malaria transmissions as well as sudden epidemics have been the peculiar characteristics of the districts. Thus, decision makers and health managers need to maintain the quality and intensity of interventions to prevent the cyclic resurgence of epidemics. The earlier epidemic in the study area could be part of the larger malaria epidemic reported nationwide in 2003/2004[4]. The significant drop in malaria incidence after explosive epidemics may be due to the impact of control measures intensively implemented, such as insecticide-treated nets (ITNs), indoor residual spraying (IRS) and other vector control methods [26], or it might be due to the effect of climatic conditions unfavorable for the survival of the vectors, or the proportions of susceptible population might be decreased. 

The incompleteness and non-representativeness of malaria data could underestimate the actual malaria transmission in the study area. Using malaria cases drawn from monthly malaria reports, and estimating the space-time variation of malaria at a district levels could be important and add value in malaria prevention and control programs in spite of the size of available data. 

The spatial scan statistics are quite useful and the most popular cluster detection techniques, and an important additional tools for evaluating disease clusters and early detection of disease outbreaks using routinely collected and available data[27]. SaTScan is a vigorous software package used to detect, analyze, and characterize the spatial and temporal pattern of malaria clusters in recent years [5,9,10,12]. The limitations are clusters that are not similar in shape to the scanning window can produce errors i.e. false inclusion and exclusion and cannot detect holes in clusters [28]. 

This study could help to understand and estimate malaria risk better. Further studies are vital to identify the main causes of bigger malaria transmission risk in the detected districts; detecting and understanding of clusters in space and time at village and individual level are important, and more detailed GIS and demographic analysis could further refine possible strategies and allow more rational choices. 

##  Conclusions

Even in small geographic areas, malaria transmission shows heterogeneity. Routinely collected data can provide useful information to guide malaria control efforts if the data are analyzed at the appropriate time using advanced statistical tools. 

## References

[1] Yeshiwondim AK, Gopal S, Hailemariam AT, Dengela DO, Patel HP (2009) Spatial analysis of malaria incidence at the village level in areas with unstable transmission in Ethiopia. Int J Health Geogr: 8: 5. doi:10.1186/1476-072X-8-5. PubMed: 19171051. 19171051 PMC2646707

[2] Fontaine RE, Najjar AE, Prince JS (1961) The 1958 malaria epidemic in Ethiopia. Am J Trop Med Hyg 10: 795-803. PubMed: 13893940. 13893940 10.4269/ajtmh.1961.10.795

[3] FMOH (2012) President’s Malaria Initiative Malaria Operational Plan (Mop) Ethiopia.

[4] Negash K, Kebede A, Medhin A, Argaw D, Babaniyi O et al. (2005) Malaria epidemics in the highlands of Ethiopia. East Afr Med J 82: 186-192. PubMed: 16122086. 16122086 10.4314/eamj.v82i4.9279

[5] Gaudart J, Poudiougou B, Dicko A, Ranque S, Toure O et al. (2006) pace-time clustering of childhood malaria at the household level: a dynamic cohort in a Mali village. BMC Public Health: 6: 286. doi:10.1186/1471-2458-6-286. PubMed: 17118176. 17118176 PMC1684261

[6] Bousema T, Griffin JT, Sauerwein RW, Smith DL, Churcher TS et al. (2012) Hitting Hotspots: Spatial Targeting of Malaria for Control and Elimination. PLOS Med 9(1): e1001165 PubMed: 22303287. 22303287 10.1371/journal.pmed.1001165 PMC3269430

[7] Kleinschmidt I, Sharp B, Mueller I, Vounatsou P (2002) Rise in malaria incidence rates in South Africa: small area spatial analysis of variation in time trends. Am J Epidemiol: 155: 257-264. doi:10.1093/aje/155.3.257. PubMed: 11821251. 11821251

[8] Chowell G, Munayco CV, Escalante AA, McKenzie FE (2009) The spatial and temporal patterns of falciparum and vivax malaria in Perú: 1994–2006. Malar J 8: 142. doi:10.1186/1475-2875-8-142. PubMed: 19558695. 19558695 PMC2714521

[9] Coleman M, Coleman M, Mabuza AM, Kok G, Coetzee M et al. (2009) Using the SaTScan method to detect local malaria clusters for guiding malaria control programmes. Malar J 8: 68. doi:10.1186/1475-2875-8-68. PubMed: 19374738. 19374738 PMC2679049

[10] Haque U, Huda M, Hossain A, Ahmed SM, Moniruzzaman M et al. (2009) Spatial malaria epidemiology in Bangladeshi highlands. Malar J 8: 185. doi:10.1186/1475-2875-8-185. PubMed: 19653914. 19653914 PMC2732922

[11] Hui F-M, Xu B, Chen Z-W, Cheng X, Liang L et al. (2009) Spatio-Temporal Distribution of Malaria in Yunnan Province, China. Am J Trop Med Hyg 81(3): 503-509. PubMed: 19706922. 19706922

[12] Mboera LEG, Senkoro KP, Mayala BK, Rumisha SF, Rwegoshora RT et al. (2010) Spatio-temporal variation in malaria transmission intensity in five agro-ecosystems in Mvomero district, Tanzania. Geospatial Health 4(2): 167-178. PubMed: 20503186. 20503186 10.4081/gh.2010.198

[13] Wen L, Li C, Lin M, Yuan Z, Huo D et al. (2011) Spatio-temporal analysis of malaria incidence at the village level in a malaria-endemic area in Hainan, China. Malar J: 10: 88. doi:10.1186/1475-2875-10-88. PubMed: 21492475. 21492475 PMC3094226

[14] Peterson I, Borrell LN, El-Sadr W, Teklehaimanot A (2009) Temporal-Spatial Analysis of Malaria Transmission in Adama, Ethiopia. Am J Trop Med Hyg 81(6): 944-949. doi:10.4269/ajtmh.2009.08-0662. PubMed: 19996421. 19996421

[15] Yeshiwondim AK, Gopal S, Hailemariam AT, Dengela DO, Patel1 HP (2009) Spatial analysis of malaria incidence at the village level in areas with unstable transmission in Ethiopia. International Journal of Health Geographics 8:5 10.1186/1476-072X-8-5 PMC2646707 19171051

[16] Abeku TA, van Oortmarssen GJ, Borsboom G, de Vlas SJ, Habbema JDF (2003) Spatial and temporal variations of malaria epidemic risk in Ethiopia: factors involved and implications. Acta Tropica 87: 331-340. 12875926 10.1016/s0001-706x(03)00123-2

[17] Jackson MC, Huang L, Luo J, Hachey M, Feuer E (2009) Comparison of tests for spatial heterogeneity on data with global clustering patterns and outliers. Int J Health Geogr 8: 55. doi:10.1186/1476-072X-8-55. PubMed: 19822013. 19822013 PMC2770045

[18] http://www.csa.gov.et.

[19] FDREMoH (2009) ederal Democratic Republic of Ethiopia Ministry of Health: National Strategic Plan for Malaria Prevention, Control and Elimination in Ethiopia, 2011-2015 Addis Ababa, Ethiopia; 2009.

[20] Teklehaimanot HD, Lipsitch M, Teklehaimanot A, Schwartz J (2004) Weather-based prediction of Plasmodium falciparum malaria in epidemic-prone regions of Ethiopia I. Patterns of lagged weather effects reflect biological mechanisms. Malar J 3: 41. doi:10.1186/1475-2875-3-41. PubMed: 15541174. 15541174 PMC535540

[21] ESRI (2011) ArcGIS Desktop: Release 10. Redlands, CA, USA: Environmental Systems Research Institute.

[22] Kulldorff M (1997) A spatial scan statistic. Commun Statist Theory Methods 26: 1481-1496. doi:10.1080/03610929708831995.

[23] Kulldorff M, SaTScan TM (2009). ser Guide for version 9.0. Available: http://www.satscan.org. Accessed 2012 November 21.

[24] Kulldorff M (1999) An isotonic spatial scan statistic for geographical disease surveillance. Journal of the National Institute of Public Health: 48:94-101.

[25] Kulldorff M, Nagarwalla N (1995) Spatial disease clusters: detection and inference. Statist Med 14: 799-810. doi:10.1002/sim.4780140809. PubMed: 7644860. 7644860

[26] Jima D, Getachew A, Bilak H, Steketee RW, Emerson PM et al. (2010) Malaria indicator survey 2007, Ethiopia: coverage and use of major malaria prevention and control interventions. Malar J 9: 58. doi:10.1186/1475-2875-9-S2-P58. PubMed: 20178654. 20178654 PMC2841196

[27] Kulldorff M, SaTScan TM (2009). ser Guide for version 9.0. Available: http://www.satscan.org.

[28] Robertson C, Nelson TA (2010) Review of software for space-time disease surveillance. Int J Health Geogr 9: 16. doi:10.1186/1476-072X-9-16. PubMed: 20226054. 20226054 PMC2848213

